# Effects of a botulinum toxin type A injection on the masseter muscle: An animal model study

**DOI:** 10.1186/s40902-015-0010-8

**Published:** 2015-02-24

**Authors:** Si-Yeok Park, Young-Wook Park, Young-Jun Ji, Sung-Wook Park, Seong-Gon Kim

**Affiliations:** 1 Department of Oral and Maxillofacial Surgery, College of Dentistry, Gangneung-Wonju National University, Gangneung, Republic of Korea; 2 Department of Oral and Maxillofacial Surgery, College of Dentistry, Gangneung-Wonju National University, Gangneung, Republic of Korea; 3 Department of Biomedical Engineering, Ulsan University, Ulsan, Republic of Korea; 4 Department of Electronic Engineering, Gangneung-Wonju National University, Gangneung, Republic of Korea; 5 Department of Oral and Maxillofacial Surgery, College of Dentistry, Gangneung-Wonju National University, Gangneung, Republic of Korea; 6 Oral and Maxillofacial Surgery, Gangneung-Wonju National University-College of Dentistry, Gangneung, 210-702 Republic of Korea

**Keywords:** Botulinum toxin type A, Electromyography, Masseter muscle, Peak voltage

## Abstract

**Background:**

The aim of this study was to investigate the effect of a botulinum toxin type A (BTX-A) injection in the masseter muscle using electromyography (EMG) in an animal model.

**Methods:**

Ten male adult (>3 months of age) New Zealand white rabbits were used. Muscle activity was continuously recorded from 8 hours before to 8 hours after BTX-A injection. The rabbits received unilateral BTX-A injections of either 5 units (group 1, n = 5) or 20 units (group 2, n = 5).

**Results:**

The masseter muscle activity of the rabbits was significantly reduced immediately after BTX-A injection (P < 0.05 for both groups). When the results from group 1 were compared with those from group 2, only the peak voltage was significantly decreased in group 2 (P = 0.013).

**Conclusion:**

Masseter muscle activity measured by EMG was immediately decreased after a BTX-A injection.

## Background

The abnormal contracture of skeletal muscle often causes pathologic problems. In the oral and maxillofacial areas, abnormal muscle action may cause temporomandibular disorder (TMD) [1], postoperative relapse after orthognathic surgery [2], and open-bite after trauma [3]. To treat TMD, muscle relaxants have been frequently prescribed [4]. To prevent postoperative relapse, muscles around the mandible are surgically stripped [5]. For the correction of open-bite, intermaxillary fixation and adaptation is commonly implemented [6]. However, the therapeutic efficacy of these treatments is not always predictable [6]. In addition, the effects of these treatments cannot be confirmed immediately after treatment initiation.

When Botulinum toxin (BTX) A is injected into masticatory muscles, it immediately causes temporary muscle paralysis, weakness and atrophy due to its pharmacological properties [7]. BTX is a biological exotoxin produced by the gram-positive anaerobic bacterium *Clostridium botulinum*. BTX causes local muscle paralysis and a reduction in overall contraction power by inhibiting the release of acetylcholine at cholinergic motor nerve terminals [8]. The bacterium produces seven serologically distinct BTXs that are potent neuroparalytic agents and are designated A-G [9]. Administration of Botulinum toxin type A (BTX-A) is considered to be safe by the U.S. Food and Drug Administration (FDA) [10], although some adverse side effects, such as systemic weakness, have been reported [11]. BTX-A has been successfully applied to various anatomical areas, especially the oral and maxillofacial regions. BTX-A is used to treat sialorrhoea, TMD, bruxism, focal dystonia, muscle spasm, and muscle hypertrophy [12-16]. In addition, the injection of BTX-A into the digastric muscle corrected post-operative anterior open bite [17]. However, there have been few animal studies about the effects of BTX on the masticatory muscle.

Electromyography (EMG) has been used extensively for diagnostic purposes, such as facial muscle localization before BTX-A injection [18-20]. However, few studies have used EMG to ascertain the effects of BTX injection on targeted muscles. This lack of information concerning muscle activity is probably due to the limitations of the available recording techniques, which require physical restraints or cable connections and thus have restricted long-term EMG studies of natural behavior. However, since the introduction of radio-telemetry, wireless transmission of EMG signals has enabled recording from freely moving animals [21]. Recently, various EMG variables were also developed to assess the masticatory muscle activity. In particular, root mean square (RMS) and peak voltage have been reported as highly reliable and reproducible indicators of masticatory muscle activity [22].

In this study, the EMG activities of rabbit masseter muscles were measured before and after BTX-A injection. The aims of this study were to investigate the immediate, dose-dependent effects of a BTX-A injection using EMG in an animal model.

## Methods

Ten male adult (>3 months of age) New Zealand white rabbits were used. This experiment was approved by the Institutional Animal Care and Use Committee of the Gangneung-Wonju National University, Gangneung, Republic of Korea (GWNU-2013-27). General anesthesia was administered by an intramuscular injection of 0.5 of mL tiletamine and zolazepam (125 mg/mL; Zoletil; Bayer Korea, Seoul, Republic of Korea) and 0.5 mL of xylazine (10 mg/kg body weight; Rompun; Bayer Korea).

**Figure 1:**
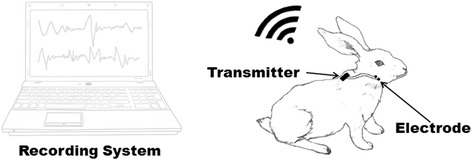
**Schematic configuration of the electromyography (EMG) recording system.**

The rabbits were implanted with electrodes for telemetrical recording of the intramuscular EMG of the masseter muscle. The transmitter was placed in the shoulder, and bipolar electrodes were placed subcutaneously and led to an incision in the right submandibular region that was used for recording the muscle potentials. The electrodes were inserted into the masseter muscles and sutured at the muscle surfaces to prevent them from dislodging. Figure 1 shows a schematic configuration of the EMG measurement setup. The electrode signal was digitized at a sampling rate of 500 Hz and was wirelessly transmitted to a computer.

**Figure 2:**
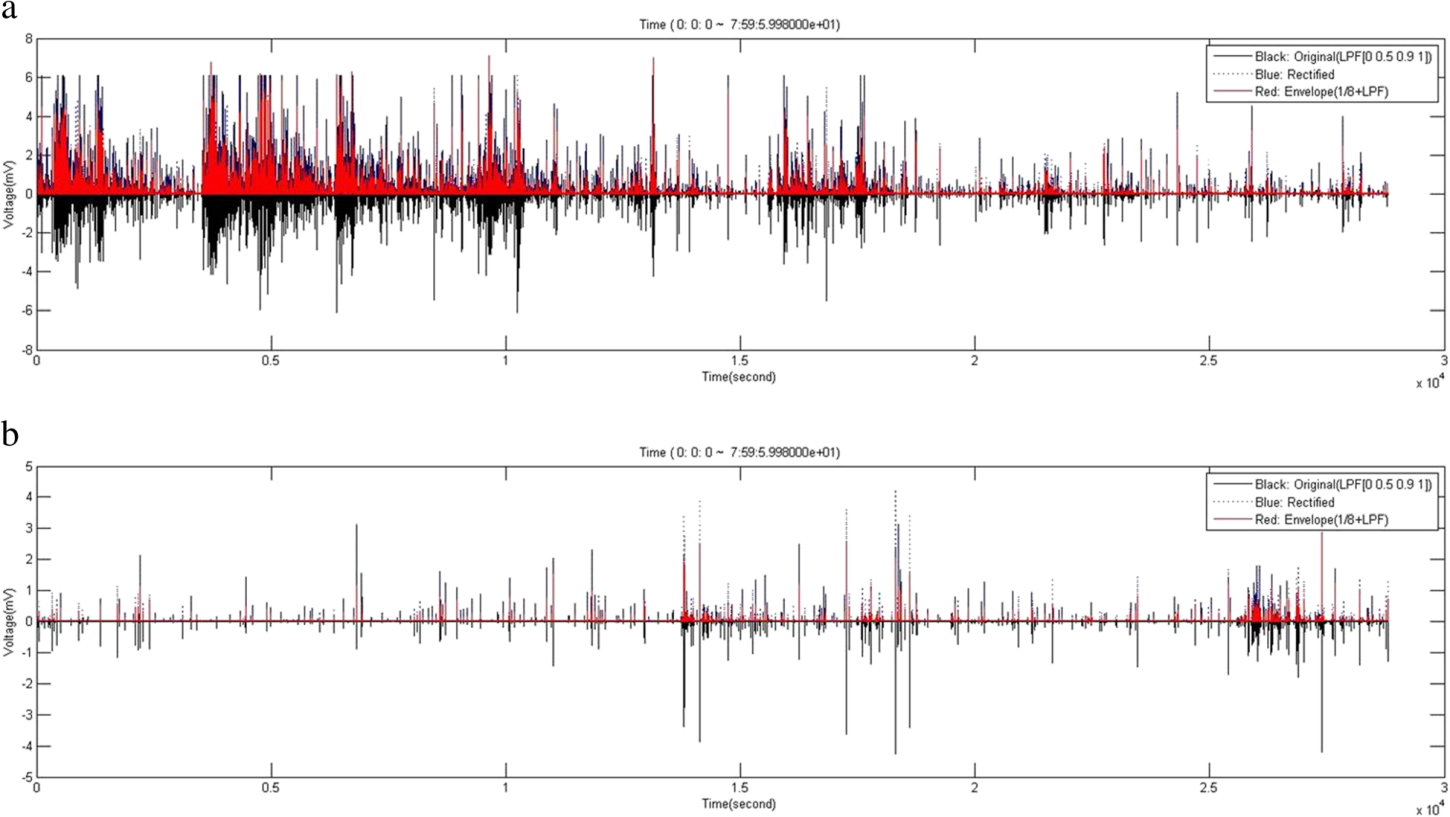
**Raw electromyography (EMG) signals from the rabbit masseter muscles. a**. EMG signals before BTX-A injection. **b**. EMG signals after BTX-A 20U injection.

Muscle activity was continuously recorded for 8 hours, starting 12 hours after surgery when the animals had regained normal feeding behavior (Figure 2a). BTX-A was purchased from company (Meditox, Cheongwon, Korea). The amount of BTX-A to the rabbit was referenced from previous publication [23] and modified in high concentration. One day following surgery, the rabbits received a unilateral masseter injection of either 5 units (group 1, n = 5) or 20 units of BTX-A (group 2, n = 5). BTX-A was diluted with normal saline as 5 unit/ 0.1 ml. BTX-A was injected into the middle of the masseter muscle. Muscle activity was recorded immediately thereafter for 12 hours (Figure 2b).

The acquired signals were processed using MATLAB® (MathWorks Inc., Natick, Massachusetts, USA) to calculate the relevant parameters. The following two parameters are shown for each EMG signal: 1) Peak voltage from the rectified signal and 2) RMS value from the original signal.

The recorded data were analyzed using SPSS software (SPSS, Inc., Chicago, Illinois, USA). The differences between the EMG values before and after injection were analyzed with paired t tests. The differences between groups were evaluated with independent sample t tests. The significance level for all tests was p < 0.05.

## Results

A summary of the recorded values is shown in Table 1. In group 1, the peak voltage before injection was 6.42 ± 0.50 mV and 4.73 ± 1.25 mV after injection. The difference before and after injection was statistically significant (P = 0.044). In group 1, the RMS before injection was 0.28 ± 0.07 mV and 0.12 ± 0.05 mV after injection. The difference between before and after injection was also statistically significant (P = 0.002). In group 2, the peak voltage was 6.49 ± 0.63 mV and 2.70 ± 0.98 mV before and after injection, respectively, and this difference was statistically significant (P < 0.001). In group 2, the RMS was 0.31 ± 0.15 mV and 0.11 ± 0.10 mV before and after injection, respectively, and this difference was also statistically significant (P = 0.001).

**Table 1:** **Summary of results**

		**Pre-injection**	**Post-injection**	**P-value**
Group 1	Peak voltage	6.42 ± 0.50 mV	4.73 ± 1.25 mV	0.044
	RMS	0.28 ± 0.07 mV	0.12 ± 0.05 mV	0.002
Group 2	Peak voltage	6.49 ± 0.63 mV	2.70 ± 0.98 mV	<0.001
	RMS	0.31 ± 0.15 mV	0.11 ± 0.10 mV	0.001

RMS: root mean square, Group 1: BTX-A 5 unit treated group, Group 2: BTX-A 20 unit treated group.

**Figure 3:**
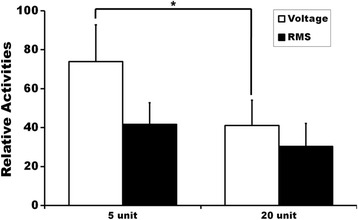
**Comparison between group 1 (5U) and group 2 (20U).** The difference in peak voltage relative activity between groups was statistically significant (P = 0.013).

When group 1 was compared with group 2, the relative peak voltage activity was 73.93 ± 18.88% and 41.14 ± 12.96%, respectively (Figure 3), and this difference between groups was statistically significant (P = 0.013). Additionally, the relative RMS activities in groups 1 and 2 were 41.68 ± 11.06% and 30.38 ± 11.84%, respectively (Figure 3), but this difference was not statistically significant (P > 0.05).

## Discussion

BTX-A has been widely used for cosmetic and therapeutic purposes [24]. BTX-A causes chemical denervation and the paralysis of associated muscles by preventing the release of membrane-bound acetylcholine at the neuromuscular junction [25]. In this study, we demonstrated that the rabbit masseter muscle showed reduced peak voltage and RMS activity as measured EMG immediately after BTX-A injection. There are several methods to treat abnormal muscle contracture. Among them, very few studies have used EMG to evaluate the effects of BTX-A injection.

RMS and peak voltage are indicators of masticatory muscle activity [22]. After BTX-A injection, all of the EMG parameters were immediately decreased. Regarding peak voltage, both groups 1 and 2 exhibited significantly lower values when compared with pre-injection measurements. Peak voltage is generally regarded as the maximum muscle power of the measured muscle [26,27]. Therefore, when the peak voltage is reduced, maximum muscle power may also be decreased [28]. The decreased peak voltage might be due to the paralytic effects of BTX-A. RMS also showed similar results in that groups 1 and 2 RMS values were significantly different after injection. RMS measurements indicate the activity of the masseter muscle, and therefore, decreased RMS values suggest that masseter muscle activity was also reduced.

Group 2 had lower mean values for all of the parameters tested than did group 1. However, only the peak voltage of group 2 was significantly lower than that of group 1 (P = 0.013). Generally, the paralytic effect of BTX-A is dose dependent [29]. However, the local effects of BTX-A plateau at a certain dose beyond which increased BTX-A administration does not add therapeutic benefit [29]. Instead, the risk of systemic complications will be increased at such a dose. For the treatment of masseteric muscle hypertrophy, 5 units of BTX-A are injected into patients and a total of 25–30 units are administered on each side [30]. When group 1 was compared with group 2, the relative peak voltage activity was 73.93 ± 18.88% and 41.14 ± 12.96%, respectively (Figure 3). Though 20 units of BTX-A was 4-fold greater dose than the 5-unit BTX-A injection, the peak voltage values were reduced by only 30% in group 2 compared to group 1. Regarding the RMS values, the difference between groups was not statistically significant. Therefore, injecting increased doses of BTX-A into a single point might not have much value.

Factors such as time to onset of action are important to patients. However, there is limited information published concerning the time to onset for BTX-A treatment. The BTX-A-injected masseter muscles showed significantly lower average activity than did uninjected muscles at weeks 1 and 2 after injection [31]. Muscle activity remained low at least through week 10 in the injected muscle. The peak paralytic effects of BTX-A have been reported at 2 to 3 weeks post-injection, and muscular function typically starts to return by 3 months [31]. Additionally, masseter-induced bite force was dramatically decreased by 85% at week 3 and 65% at week 7 compared with controls [32]. However, the onset time of muscle weakness has been unclear in previous reports. In this study, the reduced EMG activities were observed immediately after the BTX-A injection. Therefore, BTX-A injection in patients with undesirable muscle activity may produce immediate therapeutic effects. In fact, a patient with post-traumatic open-bite could close his occlusion immediately after BTX-A injection [17].

BTX-A injection could also be considered for patients who receive orthognathic surgery to prevent post-operative relapse. Most postoperative relapse in orthognathic surgery occurs within 2 months post-surgery [33]. In another study, the EMG activity of the treated muscle remained low for at least 2 months, and then, activity slowly recovered. However, even after 3 months, the activity levels remained lower than those measured before injection [34]. Therefore, BTX-A injection might be helpful in preventing post-operative relapse in patients who received orthognathic surgery. However, this assumption requires further clinical study.

Our animal study had some limitations. Although the EMG was recorded for 8 hours before BTX injection as the baseline EMG activity, the bite patterns of the animals were not consistent. Additionally, the number of bites observed after BTX injection was decreased. The decreased biting activity following the BTX injection may be due to stress after the injection. Although the implanted electrode had greater positional stability than surface electrodes used in other animal experiments, it was not free from movement. These limitations may be overcome in a human study with a similar experimental setting.

## Conclusion

In conclusion, rabbit masseter muscle activity was immediately reduced after BTX-A injection. When 5-unit BTX-A injections were compared with 20-unit injections, only the peak voltage was significantly decreased in the animals that received the greater dose.

## Abbreviations

BTX-A: Botulinum toxin type A
EMG: Electromyography
TMD: Temporomandibular disorder
FDA: Food and Drug Administration
RMS: Root mean square
